# FS-93, an Hsp90 inhibitor, induces G2/M arrest and apoptosis via the degradation of client proteins in oncogene addicted and derived resistant cancer cells

**DOI:** 10.18632/oncoscience.156

**Published:** 2015-04-22

**Authors:** Liping Zhang, Aijun Shen, Lu Wang, Hongchun Liu, Danqi Chen, Bing Xiong, Jingkang Shen, Meiyu Geng

**Affiliations:** ^1^ Division of Anti-tumor Pharmacology, State Key Laboratory of Drug Research, Shanghai Institute of Materia Medica, Chinese Academy of Sciences, Shanghai, China; ^2^ Synthetic Organic and Medicinal Chemistry Laboratory, Shanghai Institute of Materia Medica, Chinese Academy of Sciences, Shanghai, China; ^3^ School of Medicine and Pharmacy, Ocean University of China, Shandong, China

**Keywords:** hsp90, oncogene addiction, resistance, G2/M arrest, apoptosis

## Abstract

Inhibition of heat shock protein 90 (Hsp90) abrogates signaling of multiple aberrantly activated oncogenic proteins simultaneously, particularly mutated or amplified kinases, which provides an attractive approach for cancer treatment. Here, we described that FS-93, a potent Hsp90 inhibitor, impacted the survival of several types of oncogene addicted cancer cells through inducing G2/M arrest and apoptosis. Mechanistically, FS-93 treatment triggered the degradation of key client proteins such as HER2, EML4-ALK and c-Met and thereby abolished their downstream signaling pathways. Importantly, FS-93 alone circumvented MET amplification contributed acquired resistance to EGFR inhibition. Our study implicates that targeting Hsp90 is a promising alternative therapeutic tactic in oncogene addicted and derived resistant cancer cells.

## INTRODUCTION

Heat shock protein 90 (Hsp90) is a highly conserved and constitutively expressed molecular chaperone that facilitates the folding of client proteins and regulates their stability [1-3]. Hsp90 is ubiquitously overexpressed in cancer cells and comprises a multi-chaperone active complex in contrast to its latent state in normal cells [4-7]. Many client proteins of Hsp90, such as HER2, c-Met, EGFR, BCR-ABL, AKT and RAF1, et.al, are believed to play essential roles in cell survival, invasion, angiogenesis and other characteristic properties of cancer cells [8-12]. Upon inhibition of Hsp90, multiple oncogenic signaling cascades can be attenuated effectively, which makes Hsp90 a promising target for cancer therapy [13, 14]. Tremendous preclinical evidence has proved the rationale for targeting Hsp90 and dozens of Hsp90 inhibitors, including 17-DMAG, IPI-504, NVP-AUY922, BIIB021 and STA-9090, have been discovered accordingly and are currently in phase I-III clinical trials across multiple tumor types [15-17].

In contrast to other well defined kinase inhibition such as EGFR mutation for gefitinib and EML4-ALK fusion for crizotinib, it's still not clear whether the effect of Hsp90 inhibitors in clinic results from the degradation of a more sensitive client protein or general effects on multiple clients [18-22]. However, some clinical and experimental investigations have indicated that Hsp90 inhibitors are most effective in oncogene addicted tumor types where the survival of individual cancer particularly depends on specific driving oncogenes [8, 10, 23, 24]. Of note, oncogene addicted cancer cells often override their initial response to target inhibition and end up in acquired resistance inevitably stemmed from re-activation of compensatory oncogenes [25-28]. Given its ability to shut off multiple oncogenic signaling pathways simultaneously, targeting Hsp90 has exhibited its advantage to delay and circumvent the resistance acquisition than routine mechanism based combinatorial strategies.

**Figure 1:**
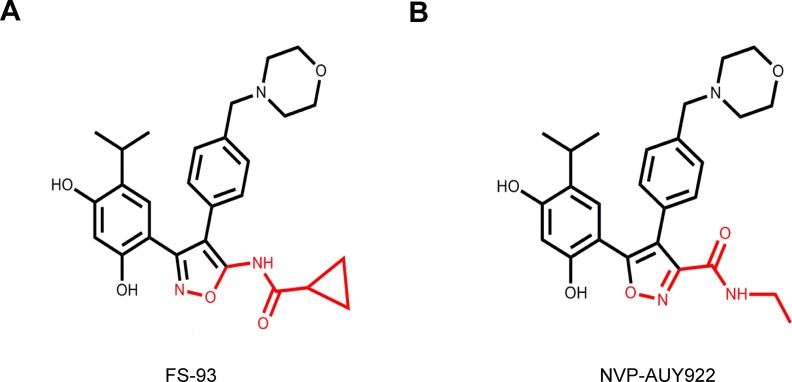
Chemical structures of FS-93 and NVP-AUY922 **A.** FS-93. **B.** NVP-AUY-922.

We have reported that FS-93, an analog of NVP-AUY922, is a potent small-molecule inhibitor of Hsp90 which based on the 4, 5-diarylisoxazole scaffold [29] (Figure 1). The preliminary data demonstrated that FS-93 inhibited the proliferation of many cancer cell lines, in which EGFR mutation addicted HCC827 cells showed exquisite sensitivity. This observation implied that oncogene addicted cancer cells should be selected for further preclinical investigation. Hence, in this study, we aimed to evaluate the pharmacological effects and underlying molecular mechanisms of FS-93 in oncogene addicted cancer cells. We discovered that FS-93 treatment impaired the survival of several types of oncogene addicted cancer cells through degrading driving oncogenes, which led to G2/M phase arrest and apoptosis. In addition, FS-93 alone circumvented gefinitib derived acquired resistance via destabilizing co-addicted EGFR and c-Met concurrently.

## RESULTS

### FS-93 impacts the survival of oncogene addicted cancer cells through degrading driving onco-proteins

**Figure 2:**
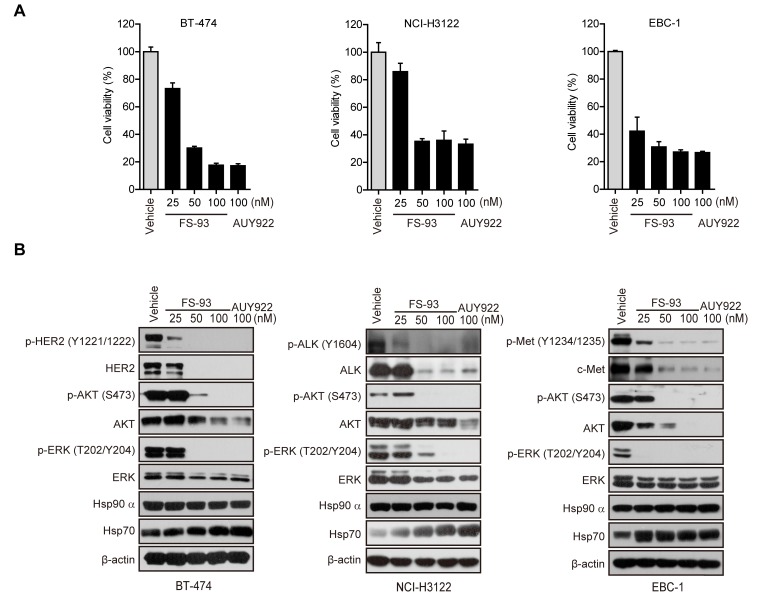
FS-93 modulates the proliferation of oncogene addicted cancer cells through destabilizing oncogenic kinases **A.** Effects of FS-93 on cell proliferation. BT-474, NCI-H3122 and EBC-1 cells were treated with FS-93 at 25, 50 and 100nM or NVP-AUY922 at 100 nM for 72 h. Cell viability was assessed with SRB assay. Bars represent means±SD. **B.** Effects of FS-93 on degradation of client onco-proteins. BT-474, NCI-H3122 and EBC-1 cells were treated with FS-93 at 25, 50 and 100nM or NVP-AUY922 at 100 nM for 24 h. Cell lysates were prepared and analyzed with immunoblotting.

The concept of “oncogene addiction” has proved its rationale to set the stage for molecularly targeted cancer therapy. Many cancer cells addicted oncogenes such as mutant EGFR, amplified HER2 and fused EML4-ALK rely on Hsp90 for their conformational maturation. In this study, we focused our research on evaluating the effect of FS-93 in several types of oncogene addicted cancer models. To this end, oncogene addicted cancer cell lines including BT-474 (HER2 amplification), EBC-1 (MET amplification) and NCI-H3122 (EML4-ALK fusion) were selected to test the pharmacological efficacy of FS-93. Cells were treated with indicated concentrations of FS-93 and NVP-AUY922 for 72 h and cell viability was determined using SRB assay. As shown, FS-93 treatment significantly inhibited the proliferation of BT-474, NCI-H3122 and EBC-1 cells, showing comparative effect with the positive control NVP-AUY922 (Figure 2A).

To explore whether the suppressed cell proliferation was the functional consequence of inhibiting Hsp90 directly, we investigated the expression of Hsp90 client proteins which are oncogenes crucial for the growth of indicated cells. BT-474 cells were exposed to increased concentrations of FS-93 for 24 h and the phosphorylation and basal expression of various client proteins and heat shock proteins were determined by western blotting. As expected, molecular co-chaperone Hsp70 was induced effectively and accordingly the expression of HER2 and its downstream signaling, namely the phosphorylation of AKT and ERK, were reduced after treatment of FS-93. Similarly, the degradation of ALK and c-Met, along with impaired downstream signaling, were also observed in oncogene addicted NCI-H3122 and EBC-1 cells (Figure 2B).

Together, these results indicated that FS-93 effectively inhibits the proliferation of oncogene addicted cancer cells through degrading their driving oncogenes.

### FS-93 induces G2/M phase arrest through decreasing the expression of Cdc2 and Cdc25c

**Figure 3:**
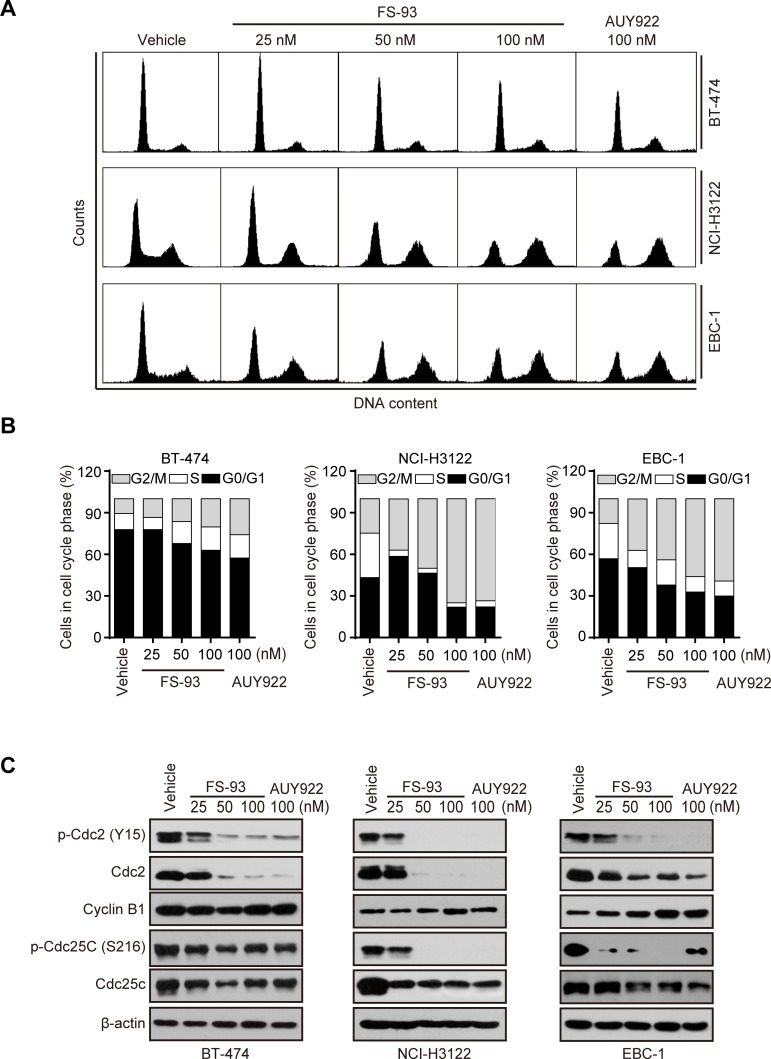
FS-93 induces G2/M cell cycle arrest in oncogene addicted cancer cells **A**., **B.** Effects of FS-93 on cell cycle distribution. BT-474, NCI-H3122 and EBC-1 cells were treated with FS-93 at 25, 50 and 100nM or NVP-AUY922 at 100 nM for 24 h. Cell cycle distribution was analyzed by FACS after propidium iodide staining. Representative images **A.** and quantification results **B.** were presented. Bars represent means±SD. **C.** Impacts of FS-93 on G2/M transition regulators. BT-474, NCI-H3122 and EBC-1 cells were treated with FS-93 at 25, 50 and 100nM or NVP-AUY922 at 100 nM for 24 h and cells lysates were immunoblotted with the indicated antibodies.

To elucidate the mechanisms of its anti-proliferative effect in oncogene addicted cancer cells, we exposed these cells to increased concentrations of FS-93 for 24 h and determined the cell cycle distribution by flow cytometry. As shown, all these cell lines tested exhibited apparent G2/M phase arrest after exposure to FS-93 (Figure 3A, 3B).

To further explore the molecular mechanisms involved in FS-93 induced G2/M phase arrest, the regulators of G2/M transition were probed after FS-93 treatment. Obviously, we found that FS-93 treatment caused universal reduction of both phosphorylation and basal expression of Cdc2 and Cdc25c in a concentration dependent manner in tested cells (Figure 3C). These data suggested that FS-93 induces G2/M phase arrest in oncogene addicted cancer cells through inhibition of the expression of Cdc2 and Cdc25c.

### FS-93 promotes apoptosis via caspase-3 dependent pathway

**Figure 4:**
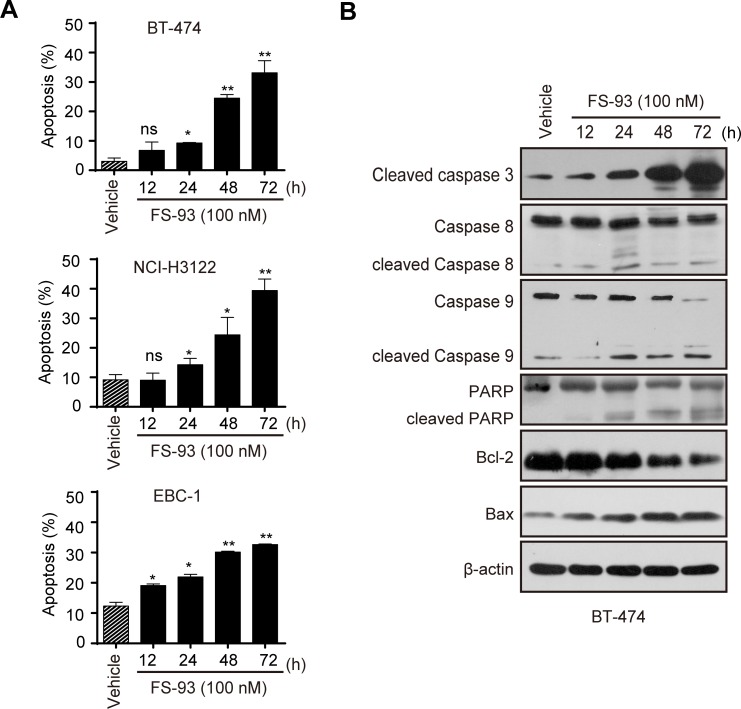
FS-93 induces apoptosis in oncogene addicted cancer cells **A.** Effects of FS-93 on apoptosis induction. BT-474, NCI-H3122 and EBC-1 cells were treated with DMSO or FS-93 at 100 nM for 12, 24, 48 and 72h. Apoptosis was determined with annexin-V/PI assay. Bars represent means±SD. **B.** Impacts of FS-93 on apoptotic proteins. BT-474 cells were treated with DMSO or FS-93 at 100 nM for 12, 24, 48 and 72h and analyzed by immunoblotted with the indicated antibodies.

We also examined whether FS-93 can induce apoptosis in tested cell lines. To this end, cells were treated with FS-93 at 100 nM for 0, 12, 24, 48 and 72 h and apoptosis was determined using annexin V/PI assay. Remarkable apoptosis was observed after treatment of FS-93 for 48 h and 72 h, where approximately 30% to 40% cells were apoptotic in cell lines tested (Figure 4A).

We then took BT-474 as an example to evaluate the pathways involved in FS-93 triggered apoptosis. BT-474 cells were treated with 100 nM of FS-93 for indicated time points. As expected, FS-93 induced a time-dependent cleavage of PARP which is a hallmark of apoptosis. Meanwhile, activation of caspase-3 was observed in a time-dependent manner. We also measured the expression of Bcl-2 family proteins termed as Bcl-2 and Bax and revealed that Bax was up-regulated while Bcl-2 was down-regulated effectively (Figure 4B).

Taken together, these results suggested that FS-93 induces apoptosis in oncogene addicted cancer cells by regulating the expression of Bcl-2 family proteins and triggering caspase-3 dependent apoptosis pathway.

### FS-93 circumvents MET amplification contributed resistance to EGFR inhibition

**Figure 5:**
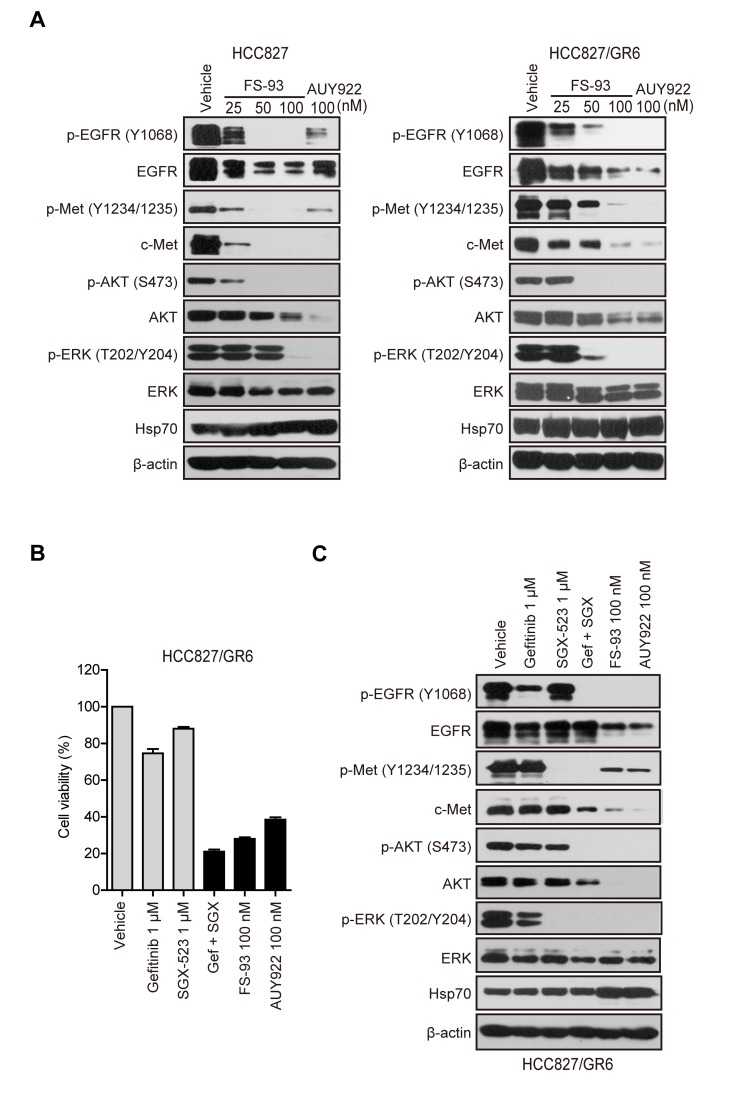
FS-93 overcomes acquired resistance of EGFR inhibition **A.** Effects of FS-93 on degradation of client onco-proteins. HCC827 and HCC827/GR6 cells were treated with FS-93 at 25, 50 and 100nM or NVP-AUY922 at 100 nM for 24 h and analyzed by immunoblotting. **B.** Effects of FS-93 on cell growth. HCC827/GR6 cells were exposed to DMSO, gefitinib at 1 μM, SGX-523 at 1 μM, their combination, FS-93 at 100 nM or NVP-AUY922 at 100 nM for 72h. Cell viability was determined with SRB assay. Bars represent means±SD. **C.** Effects of FS-93 on the modulation of signaling pathways. HCC827/GR6 cells were exposed to DMSO, gefitinib at 1 μM, SGX-523 at 1 μM, their combination, FS-93 at 100 nM or NVP-AUY922 at 100 nM for 24h and analyzed with immunoblotting.

Although the clinical application of kinase inhibitors has been proved promising, the major challenge lies in quickly occurred acquired resistance which often stems from activated compensatory kinases. Intriguingly, growing bodies of evidence has suggested the potential contribution of circumvent resistance by targeting Hsp90 [30-32]. Thus, we took EGFR inhibitor gefitinib resistant HCC827/GR6 cells, which co-addicted to both EGFR mutation and MET amplification, as an example to determine the ability of FS-93 to overcome acquired resistance of kinase inhibitors. After treatment with increased concentrations of FS-93 for 24 h in HCC827/GR6 cells, the expression of both EGFR and c-Met was diminished significantly and led to the abrogation of downstream AKT and ERK activity, similar to that of their parental HCC827 cells which only rely on mutant EGFR for survival (Figure 5A).

As previously described, survival of HCC827/GR6 cells can be suppressed by combined inhibition of EGFR and c-Met, we then turned to compare the effect of FS-93 against this combination. HCC827/GR6 cells were treated with EGFR inhibitor gefitinib, c-Met inhibitor SGX-523, their combination and FS-93 for 72 h and cell viability was assessed. Of note, FS-93 alone apparently inhibited the proliferation of HCC827/GR6 cells to the same level of the combination of EGFR and c-Met kinase inhibitors (Figure 5B). Then, we treated HCC827/GR6 cells with indicated compounds for 24 h and examined their abilities to shut down signal transduction. As expected, FS-93 alone and the combination of gefitinib and SGX-523 both suppressed EGFR and c-Met controlled signaling pathways effectively (Figure 5C).

Collectively, these data implied that FS-93 overrides acquired resistance derived from oncogene addicted cancer cells through shutting off the re-activated compensatory signaling concomitantly.

## DISCUSSION

In this study, we investigated the mechanisms of a novel Hsp90 inhibitor FS-93 in oncogene addicted and derived resistant cancer cells. We discovered that FS-93 impacted the proliferation of cancer cells driven by different oncogenic kinases. In each model, FS-93 destabilized client proteins including amplified HER2, fused EML4-ALK, amplified c-Met and mutated EGFR respectively and abolished their downstream AKT and ERK signaling pathways, which eventually contributed to G2/M phase arrest and apoptosis. Notably, we also found that FS-93 alone thwarted MET amplification contributed acquired resistance of EGFR inhibitor gefitinib via destabilizing both EGFR and c-Met. Together, our data indicated that FS-93 is effective in both oncogene addicted and derived resistant cancer cells.

The success of predictive biomarker guided molecular targeted therapy, such as gefitinib in EGFR mutant NSCLC patients, has been witnessed in clinic.

In the case of Hsp90, however, the intrinsic features of Hsp90 inhibition makes it difficult to set the appropriate criteria for patient selection. Nonetheless, more and more evidence has implied that oncogene addicted sub-types of cancer patients might be the most responsive for Hsp90 inhibitors. Given the fact that only limited number of addicted oncogenic kinases have been identified while fewer can be targeted effectively, Hsp90 inhibition should be considered as a promising alternative approach to be applied in oncogene driving cancer sub-types those newly discovered but can't be reached by specific kinase inhibitors yet.

On the other hand, the major drawback of targeted therapies in oncogene addicted cancer cells lies in quickly acquisition of acquired resistance arises from activated compensatory kinases. In contrast to identify the detailed complementary targets, the unique character of Hsp90 inhibition provides advantages to delay and overcome acquired resistance of kinase inhibitors. This will pave a new way for the application of Hsp90 inhibitor along with the advance of kinase inhibitors utilized in clinic.

In conclusion, our study suggests that the newly developed Hsp90 inhibitor FS-93 is effective in both oncogene addicted and derived resistant cancer cells, which helps us to find an appropriate direction for further co-clinical or clinical assessment.

## MATERIALS AND METHODS

### Compounds and reagents

FS-93 was synthesized as previously described [29]. SGX-523, gefetinib and NVP-AUY922 were obtained from Selleck Chemicals (Houston, USA). All these compounds were dissolved to 10 mM with DMSO as a stock solution and stored at −20°C.

### Cell culture

Human NSCLC cell line EBC-1 was obtained from Japanese Research Resources Bank (Tokyo, Japan). Human NSCLC cell line NCI-H3122 was obtained from National Cancer Institute (Bethesda, MD). Human breast cancer cell line BT-474 was obtained from American Type Culture Collection (Manassas, VA). Human NSCLC cell line HCC827 and HCC827/GR6 were kindly gifted by Dr. Pasi A. Jänne (Dana-Farber Cancer Institute, Boston, MA). All these cell lines were authenticated by STR fingerprinting and routinely maintained in media according to the suppliers' recommendations.

### Cell proliferation assay

Cells were plated into 96-well plates at a density of 3000-8000 cells/well in triplicates. After incubation overnight, cells were exposed to indicated concentrations of compounds and incubated at 37°C for further 72 h. Cells were then fixed with 10% pre-cooled trichloroacetic acid, washed with distilled water, and stained with 4 mg/mL sulforhodamine B (SRB, Sigma, St. Louis, MO) in 1% acetic acid. SRB in the cells was dissolved in 10 mM Tris-HCl and was measured at 560 nm using spectra-MAX190 (Molecular Devices, Sunnyvale, CA). The cell proliferation inhibition rate was calculated as follows: proliferation inhibition (%) = [1−(A_560_ treated/A_560_ control)] × 100%.

### Cell cycle analysis

Cells were seeded in 6-well plates at a density of 2×10^5^ cells/well. After 24 h, the cells were treated with DMSO or indicated compounds for 24 h. Both adherent and floating cells were harvested and fixed in cold 70% ethanol for over-night at 4°C. Prior to FACS analysis, cells were centrifuged at 500 g for 5 min and washed twice with cold PBS and re-suspended in PBS containing 200 μg/ml RNase A, 50 μg/ml propidium iodide and incubated for 15 min at 37°C in the dark. Quantitation of the cell cycle distribution was evaluated using a Becton-Dickinson FACS Calibur flow cytometer (BD, San Jose, CA). Data was analyzed using Modifit LT (BD, San Jose, CA).

### Apoptosis measurement

Cells were cultured in a 6-well plate at a density of 2×10^5^ cells/well. After 24 h, the cells were treated with DMSO or indicated compounds for different timepoints. Then, the cells were trypsinized and washed once with cold PBS. Aliquots of the cells were re-suspended in 100 μL of binding buffer and stained with 5 μL of Annexin V-FITC and 5 μL of PI working solution (Vazyme Biotech, Nanjing, China) for 10 min in the dark. The cells were re-suspended in 400 μL of binding buffer and analysis was performed using a Becton-Dickinson FACS Calibur flow cytometer (BD, San Jose, CA). Data was analyzed using CELLQuest software (BD, San Jose, CA).

### Western blotting analysis

Protein extracts were prepared by washing twice in cold PBS followed by lysis with SDS-lysis buffer (50 mM Tris-HCl, pH7.4, 2%SDS). Cell lysates were boiled for 10 min and cleared by centrifugation at 14,000× g for 5 min at 4°C. The supernatant was collected and subsequently resolved by SDS-PAGE and transferred to nitrocellulose membranes, probed with the appropriate primary antibodies and then incubated with horseradish peroxidase-conjugated secondary antibodies. The immunoreactive proteins were detected using an ECL plus detection reagent (Picece, Rockford, IL) and imaged by autoradiography.

Antibodies against GAPDH, Cdc2, Cyclin B1, Hsp90α and Hsp70 were from Epitomics (Burlingame, CA). Antibodies against c-Met, phospho-Met (Y1234/1235), EGFR, phospho-EGFR (Y1068), HER2, phospho-HER2 (Y1221/1222), ALK, phospho-ALK (Y1604), phospho-Cdc2 (Y15), Cdc25c, phospho-Cdc25c (S216), AKT, phospho-AKT (S473), ERK, phospho-ERK (T202/Y204), Cleaved caspase-3, Caspase-8, Caspase-9, PARP, Bcl-2, Bax and β-actin were from Cell Signaling Technology (Beverly, MA).

### Statistical analysis

Data were presented as mean ± SD, significance was determined by Student's t-test. Differences were considered statistically significant at *p < 0.05. All statistical analysis was performed using GraphPad Prism software (San Diego, CA).
